# A small‐molecule PAI‐1 inhibitor prevents bone loss by stimulating bone formation in a murine estrogen deficiency‐induced osteoporosis model

**DOI:** 10.1002/2211-5463.12390

**Published:** 2018-02-16

**Authors:** Guangwen Jin, Alkebaier Aobulikasimu, Jinying Piao, Zulipiya Aibibula, Daisuke Koga, Shingo Sato, Hiroki Ochi, Kunikazu Tsuji, Tetsuo Nakabayashi, Toshio Miyata, Atsushi Okawa, Yoshinori Asou

**Affiliations:** ^1^ Department of Orthopedics Surgery Tokyo Medical and Dental University Japan; ^2^ Department of Orthopaedic Surgery Yanbian University Hospital Yanji City Jilin Province China; ^3^ Department of Physiology and Cell Biology Tokyo Medical and Dental University Japan; ^4^ Department of Cartilage Regeneration Tokyo Medical and Dental University Japan; ^5^ Department of Molecular Medicine and Therapy United Centers for Advanced Research and Translational Medicine Tohoku University Graduate School of Medicine Miyagi Japan

**Keywords:** bone formation, osteoporosis, ovariectomy, plasminogen activator inhibitor‐1, postmenopause

## Abstract

Osteoporosis is a progressive bone disease caused by an imbalance between bone resorption and formation. Recently, plasminogen activator inhibitor‐1 (PAI‐1) was shown to play an important role in bone metabolism using PAI‐1‐deficient mice. In this study, we evaluated the therapeutic benefits of novel, orally available small‐molecule PAI‐1 inhibitor (iPAI‐1) in an estrogen deficiency‐induced osteoporosis model. Eight‐week‐old C57BL/6J female mice were divided into three groups: a sham + vehicle (Sham), ovariectomy + vehicle (OVX + v), and OVX + iPAI‐1 (OVX + i) group. iPAI‐1 was administered orally each day for 6 weeks starting the day after the operation. Six weeks of iPAI‐1 treatment prevented OVX‐induced trabecular bone loss in both the femoral bone and lumbar spine. Bone formation activity was significantly higher in the OVX + i group than in the OVX + v and Sham groups. Unexpectedly, OVX‐induced osteoclastogenesis was partially, but significantly reduced. Fluorescence‐activated cell sorting analyses indicated that the number of bone marrow stromal cells was higher in the OVX + i group than that in the OVX + v group. A colony‐forming unit‐osteoblast assay indicated enhanced mineralized nodule formation activity in bone marrow cells isolated from iPAI‐1‐treated animals. Bone marrow ablation analysis indicated that the remodeled trabecular bone volume was significantly higher in the iPAI‐1‐treated group than that in the control group. In conclusion, our results suggest PAI‐1 blockade via a small‐molecule inhibitor is a new therapeutic approach for the anabolic treatment of postmenopausal osteoporosis.

**Abbreviations**
BMSCs: bone marrow stromal cells
CFU‐ob: colony‐forming unit‐osteoblast
MSCs: mesenchymal stem cells
OVX: ovariectomy
PAI‐1: plasminogen activator inhibitor‐1
TRAP: tartrate‐resistant acid phosphatase

Bone homeostasis is balanced via bone formation and resorption. Bone formation, attributable to osteoblast activity, and bone resorption, attributable to osteoclast activity, are tightly coupled during bone remodeling. An imbalance in these activities, such as impairment of bone formation or enhanced bone resorption, results in osteoporosis.

Estrogen deficiency‐induced bone loss, characterized by increased osteoclastogenesis, is associated with the postmenopausal stage in women 1, 2. As such, many drugs have been developed to prevent loss of bone volume (BV) in postmenopausal women. Most of these drugs, including the bisphosphonates and denosumab, target bone resorption and inactivate osteoclasts 2. However, long‐term use of antiresorptive agents results in reduced bone turnover and may cause atypical femoral fractures 3, 4 or antiresorptive agent‐related osteonecrosis of the jaw 3, 5. To reserve bone health and prevent these side effects, agents that stimulate bone formation and maintain bone turn over, such as teriparatide, are currently the focus of research 2, 6.

Plasminogen activator inhibitor‐1 (PAI‐1), a serine protease inhibitor (serpin), is a 50‐kDa single‐chain glycoprotein that inhibits urokinase plasminogen activator and tissue plasminogen activator (tPA) 7. PAI‐1 is implicated in pathophysiological conditions, including wound healing, obesity, metabolic syndrome, cardiovascular disease, and cancer 7. Recently, PAI‐1 was reported to play an important role in bone metabolism via PAI‐1‐deficient mice. For example, callus formation is accelerated in a PAI‐1‐deficient murine fracture model 8. Further, PAI‐1‐deficient mice are resistant to ovariectomy (OVX)‐induced osteoporosis 9.

A small‐molecule PAI‐1 inhibitor (iPAI‐1, molecular weight 424.81) was designed based on the original lead compounds, TM5007 10 and TM5275 11. iPAI‐1 have been shown to prevent PAI‐1‐mediated pathological conditions. TM5275 administration prevents albuminuria, mesangial expansion, extracellular matrix (ECM) accumulation, and macrophage infiltration in diabetic kidneys by suppressing PAI‐1‐mediated upregulation of fibrosis and inflammation 12. PAI‐1 inhibition enhances neutrophil‐driven angiogenesis and cell‐driven revascularization by activating the proangiogenic fibroblast growth factor (FGF)‐2 and vascular endothelial growth factor (VEGF)‐A pathways 13.

In this study, we explored the therapeutic potential of iPAI‐1, a novel iPAI‐1, in an estrogen deficiency‐induced osteoporosis model. Furthermore, we examined the effect of iPAI‐1 administration on rapid bone remodeling using a murine bone marrow ablation model. We demonstrate that administration of iPAI‐1 augments bone formation *in vivo*.

## Materials and methods

### Generation and validation of iPAI‐1

The novel iPAI‐1 molecule (molecular weight, 424.81) was discovered at the United Centers for Advanced Research and Translational Medicine (ART), Tohoku University Graduate School of Medicine (Miyagi, Japan), via an extensive structure–activity relationship study with more than 450 novel derivatives having comparatively low molecular weights (400–550 g·mol^−1^) and without symmetrical structure. The iPAI‐1 molecule was designed on the basis of the original lead compound (TM5007 10) and a successfully modified version (TM5275 11). TM5007 was identified virtually via structure‐based drug design after undergoing a docking simulation that selected for compounds that fit within the PAI‐1 cleft (s3A in the human PAI‐1 three‐dimensional structure) and were accessible to insertion in the reactive center loop 10. Compounds that bind in this cleft block reactive center loop insertion and thus prevent PAI‐1 activity. Once TM5007 had been identified as a iPAI‐1 both virtually and *in vitro/in vivo*, further compounds were derived via chemical modifications to improve the pharmacokinetic properties of the inhibitor, resulting in the generation of TM5275, and later, iPAI‐1. The inhibitory activity (IC_50_) and specificity of iPAI‐1 were assessed using recombinant PAI‐1 via a chromogenic assay as previously described 10, 11. The IC_50_ of iPAI‐1 is 3.63 μm, and its specificity was confirmed by demonstrating that it does not inhibit other serpins, including antithrombin III and α2‐antiplasmin (data not shown). To evaluate iPAI‐1 pharmacokinetics, iPAI‐1 (5 mg·kg^−1^) suspended in a 0.5% carboxymethyl cellulose sodium salt solution was administered orally by gavage to male Wistar rats (CLEA Japan, Inc., Tokyo, Japan). Heparinized blood samples were collected from the vein before (0 h) and 1, 2, 6, and 24 h after oral drug administration. Plasma drug concentrations were determined on a reverse‐phase high‐performance liquid chromatography system. Maximum drug concentration time (*T*_max_), maximum drug concentration (*C*_max_), and drug half‐life (*T*_1/2_) were calculated as 2 h, 33.8 μg·mL^−1^, and 3.9 h, respectively.

### Animal experiments

All animal experiments were approved by the Institutional Animal Care and Use Committee of Tokyo Medical and Dental University. Seven‐week‐old female C57BL/6J mice were purchased from Sankyo Labo (Tokyo, Japan). The animals were given a normal rodent chow and tap water and acclimated to conditions for 1 week before surgery. The mice were then divided into three groups, including a sham + vehicle (Sham), OVX + vehicle (OVX + v), and OVX + iPAI‐1 (OVX + i) group. The iPAI‐1 was resuspended in 200 μL 0.5% carboxymethylcellulose (MP Biomedicals, Santa Ana, CA, USA) and administered orally (10 mg·kg^−1^ body weight) daily from the day after the operation and continuing for 6 weeks. All mice were sacrificed 6 weeks after the operation.

### Histological and histomorphometric analyses

Tetracycline and calcein were injected subcutaneously for bone labeling 5 and 2 days before sacrifice. Blood and bone samples were collected at sacrifice. The undecalcified sections of the third and fourth lumbar vertebrae were stained using von Kossa and tartrate‐resistant acid phosphatase (TRAP) stains, as previously described 14. We performed static and dynamic histomorphometric analyses using the OsteoMeasure Analysis System (OsteoMetrics, Decatur, GA, USA) following the nomenclature defined by the American Society for Bone and Mineral Research as previously described 15.

### Micro‐computed tomography (CT) analysis

We obtained two‐dimensional images of the distal femurs by micro‐computed tomography (CT) analysis (Comscan, Yokohama, Japan). The following three‐dimensional morphometric parameters were determined using TRI/3d‐bon software (RATOC, Tokyo, Japan). Bone morphometric analyses were performed at a region 0.2–1 mm above the distal growth plates of the femora.

### Serum osteocalcin and urinary C‐terminal telopeptide (CTX‐1)

Serum osteocalcin was measured with an osteocalcin ELISA MK127 kit (TaKaRa Shuzo, Kyoto, Japan). Urine was collected 2 weeks after the operation from all the mice. Urinary C‐terminal telopeptide 1 (CTX‐1) levels were measured with a CTX‐1 ELISA kit (Immunodiagnostic Systems, Boldon, UK).

### Bone marrow ablation

Eight‐week‐old female C57BL/6J mice were divided into three groups, including a sham + vehicle (Sham + v), OVX + vehicle (OVX + v), and OVX + iPAI‐1 (OVX + i) group. Mice underwent OVX or sham procedure at 8 weeks of age. Then, all the mice underwent bone marrow ablation operation 4 days after the first operation. At bone marrow ablation operation, mice were anesthetized and the hair removed from both hind limbs, and the bone marrow from both femurs of each animal was ablated as previously described 14. Briefly, bilateral longitudinal incisions were made on the knees of each mouse to expose the femoral condyle by dislocation of the patella. A hole was made at the intercondylar notch of the femur using a dental drill, and 0.6‐mm‐diameter Kirschner wire was inserted into the distal end of the femur to confirm completion of marrow ablation by radiography. After removal of the Kirschner wire, the dislocated patella was reposed and the skin was sutured closed. Oral administration of iPAI‐1 (10 mg·kg^−1^) was given daily starting the day after the operation. Mice were sacrificed 12 days after the surgery; femoral BV was then evaluated by micro‐CT.

### Fluorescence‐activated cell sorting

Bone marrow cells obtained from mice were layered onto a lymphocyte‐M gradient (Cedarlane Laboratories, Burlington, Ontario, Canada). After centrifugation, mononuclear cells were collected and incubated with allophycocyanin (APC)‐conjugated anti‐CD140a or FITC‐conjugated anti‐Sca‐1 for 30 min. Cells were washed twice and analyzed using a Cytomics FC500 (Beckman Coulter, Fullerton, CA, USA).

### Colony‐forming unit‐osteoblast (CFU‐ob) assay

Bone marrow cells were seeded on 6‐well plates at a concentration of 1 × 10^5^ cells per well. After incubating in alpha‐modified minimum essential medium (α‐MEM) for 2 or 3 days, the medium was changed into osteogenic induction medium (OIM) containing 100 nmol·L^−1^ dexamethasone, 10 mmol·L^−1^ beta‐glycerophosphate, and 0.05 mmol·L^−1^ l‐ascorbic acid‐2‐phosphate to stimulate osteoblastic differentiation. The medium was changed twice per week. Four weeks after osteogenic induction, colony‐forming unit‐osteoblast (CFU‐obs) were determined via 2 % alizarin red S staining (Sigma‐Aldrich, St. Louis, MO, USA).

### Statistical analysis

Results are expressed as the mean ± SD. The Steel test or Mann–Whitney *U*‐test was used to determine statistical differences; *P *≤* *0.05 was used as the criterium for statistical significance.

## Results

To determine whether iPAI‐1 prevents estrogen deficiency‐induced bone loss, iPAI‐1 (10 mg·kg^−1^) was administered orally to OVX mice every day for 6 weeks. Uterine size in OVX mice was smaller than that in Sham mice upon sacrifice. Further, body weights of the OVX mice were significantly higher than those of Sham mice regardless of iPAI‐1 administration.

**Figure 1:**
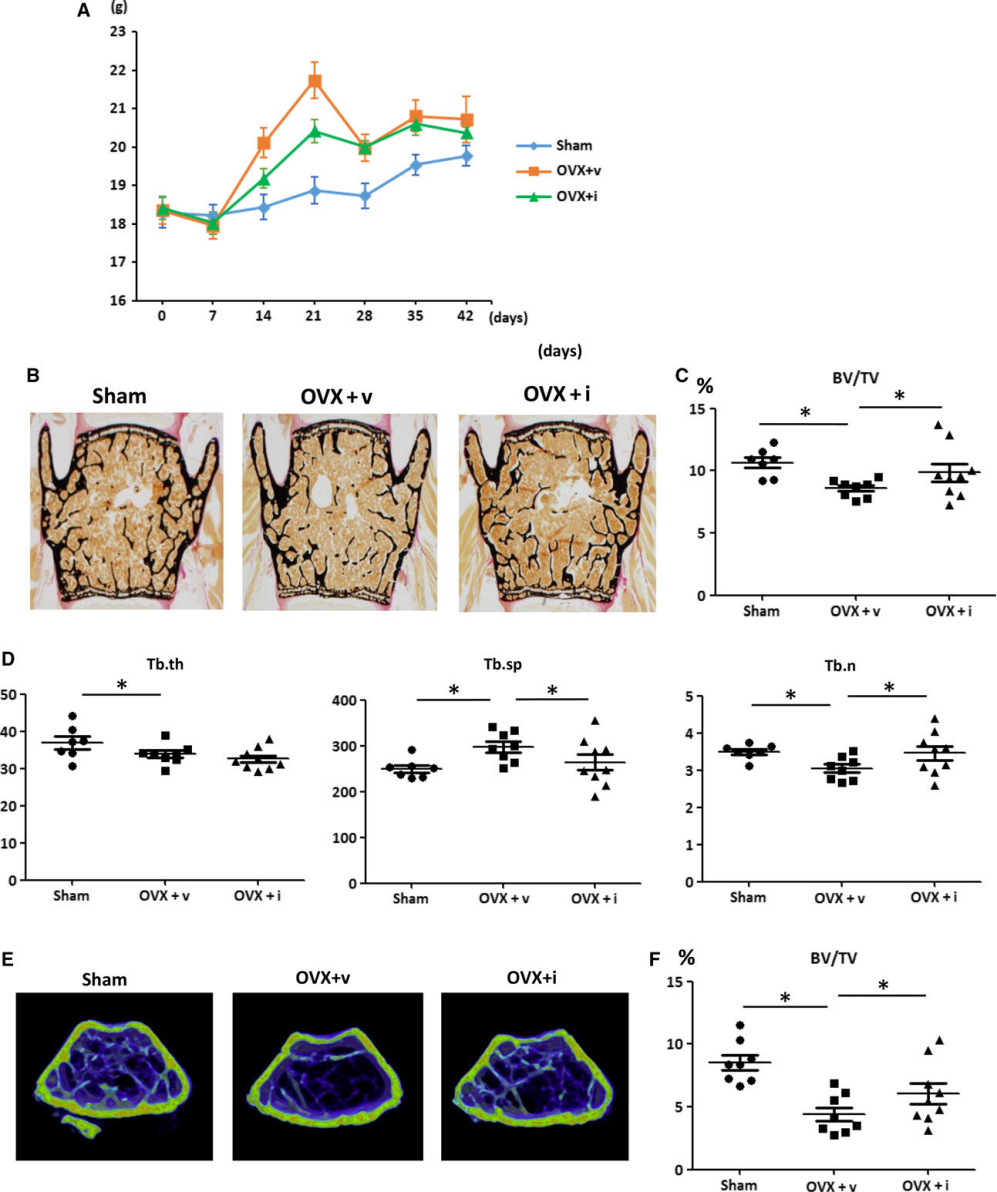
iPAI‐1 treatment in OVX mice restores trabecular BV at lumbar spine and distal femur. (A) Body weight of Sham, OVX + v, and OVX + iPAI‐1 in indicated length of time after the operation. (B–D) Trabecular bone phenotype in the mice: vertebra. (B) Representative images of lumbar spine from each of the three groups. (C–D) Histological analysis is shown of (C) trabecular BV/TV and (D) Tb.th, Tb.sp, and Tb.n in the L3 vertebra of the three groups (*n* = 8 per group). (E) Representative 3D μCT images of the distal femur region from each of the three groups. (F) Values for BV/TV. Data represent mean ± SD for eight mice/group. *P <* *0.05. OVX + v: OVX + vehicle; OVX + i: OVX + iPAI‐1.

Histological analysis indicated that trabecular BV to total volume (TV) in the vertebrae was significantly lower in OVX mice than that in Sham mice (Fig. 1B–E). Administration of iPAI‐1 to OVX mice significantly increased trabecular BV/TV. Bone histological analysis of the lumbar spine indicated that trabecular separation (Tb.sp) was higher, whereas trabecular number (Tb.n) and trabecular thickness (Tb.th) were lower in the OVX + v group than those in the Sham group. OVX + i values for Tb.sp and Tb.n returned to levels observed in the Sham group. In contrast, Tb.th in the OVX + i group was comparable to that in the OVX + v group (Fig. 1B).

To evaluate bone resorption activity, urinary levels of CTX‐1, a bone resorption marker, were determined in all mice. Mouse urine was collected 2 weeks after the operation, and the concentration of CTX‐1 was evaluated via an ELISA. As expected, CTX‐1 levels were higher in the OVX + v group than those in the Sham group. Surprisingly, the OVX + i group exhibited partial, but significant suppression of CTX‐1 concentrations. In contrast, serum Gla‐osteocalcin, a marker of bone formation, was higher in the OVX+i group than that in the Sham group (*P *<* *0.05). These results indicate iPAI‐1‐stimulated bone formation in OVX mice.

**Figure 2:**
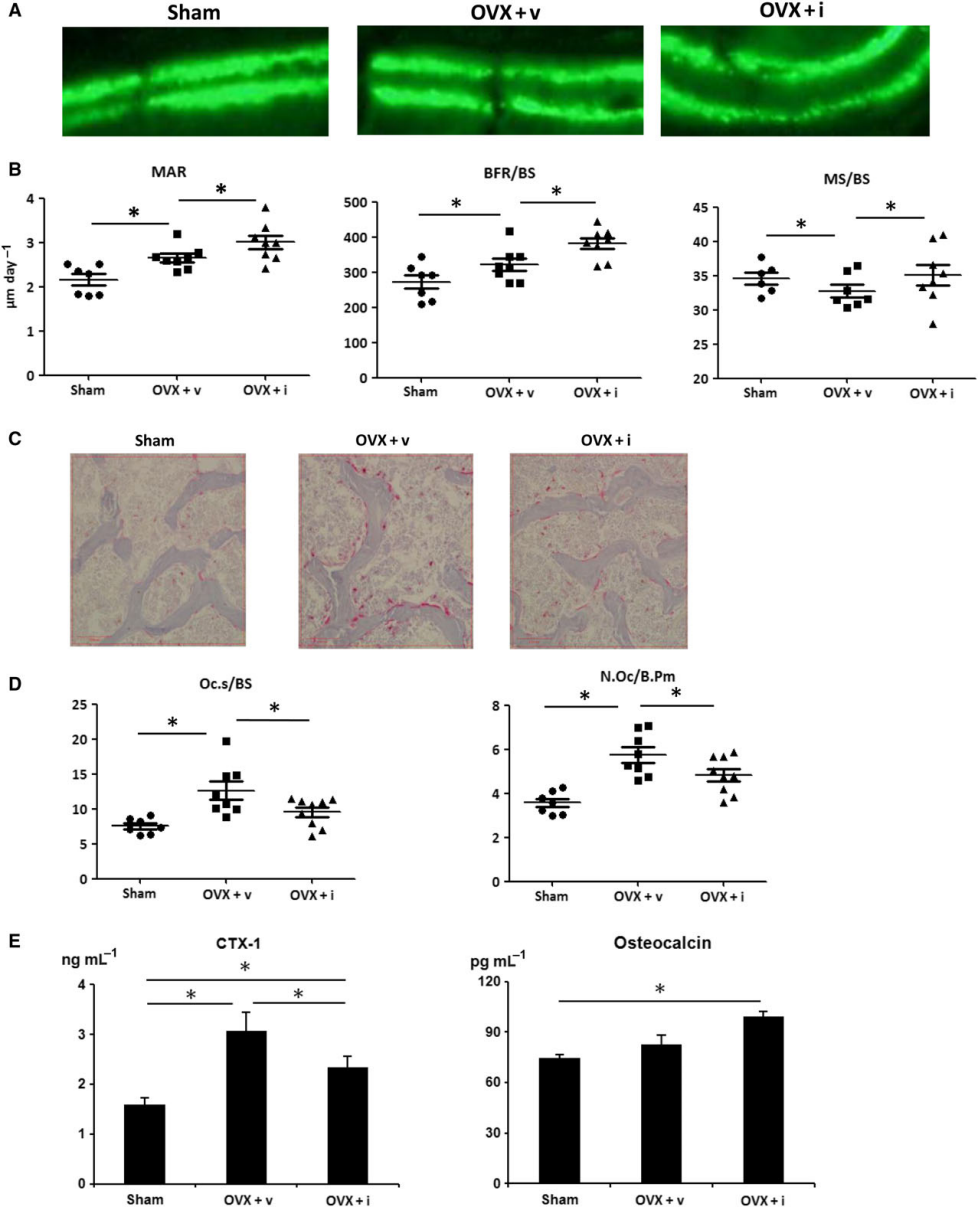
iPAI‐1 treatment increases bone formation in lumbar vertebrae as assessed by histomorphometric analysis. (A) Fluorescent micrograph of the trabecular bone section showing the calcein (green) labels. (B) MAR, BFR/BS, and MS/BS. (C) Representative images of TRAP‐stained sections from each of the three groups (scale bar, 100 μm) and (D) quantification of TRAP‐stained osteoclast surface relative to the trabecular BS and the number of osteoclasts, expressed relative to the trabecular bone perimeter (N.Oc/B.Pm). (E) Urine CTX‐1 levels. (C) Data represent mean ± SD for eight mice/group. *P <* *0.05. OVX + v: OVX + vehicle; OVX + I: OVX + iPAI‐1.

The dynamic histology of the trabecular bone in the lumbar spine was compared among the groups. OVX resulted in significant increases in the mineralizing surface to bone surface (MS/BS), bone formation rate (BFR)/BS, and mineral apposition rate (MAR) because of high bone turn over osteopenia. Consistent with the serum osteocalcin results, iPAI‐1 treatment further increased MAR and BFR/BS over that observed in the Sham and OVX groups. In agreement with the urine CTX‐1 analysis, the TRAP assay revealed a significant increase in Oc.S/BS and N.Oc per bone perimeter (B.Pm) following OVX. Administration of iPAI‐1 partially suppressed these osteoclast parameters (Fig. 2B,C).

**Figure 3:**
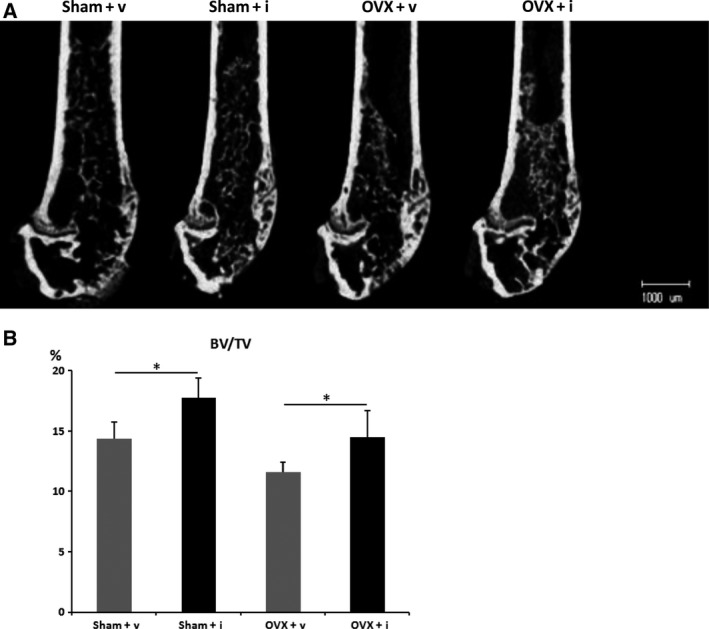
iPAI‐1 administration stimulates new bone formation *in vivo* during the repair of bone injury. (A) Micro‐CT analyses of the distal metaphyses of the femur of vehicle‐treated and iPAI‐1‐treated mice 12 days after bone marrow ablation. (B) Quantification of the newly formed bone within the ablated region of vehicle‐treated mice or iPAI‐1‐treated mice. Data represent mean ± SD for seven or eight mice/group. *P <* *0.05. Sham + v: sham operation + vehicle; Sham + i: sham operation + iPAI‐1; OVX + v: OVX + vehicle; OVX + i: OVX + iPAI‐1.

To evaluate the effect of iPAI‐1 on rapid bone remodeling, bone marrow ablation operation was performed for sham‐operated or ovariectomized mice. iPAI‐1 or the vehicle was orally administered starting the day after bone marrow ablation. BV was then evaluated 12 days after the procedure. Micro‐CT analysis indicated that the BV/TV ratio was significantly higher in the iPAI‐1‐treated group than that in the vehicle group both in sham‐operated mice and in OVX mice (Fig. 3A,B). These observations indicate that iPAI‐1 treatment stimulates bone formation and accelerates BV recovery after bone marrow ablation.

**Figure 4:**
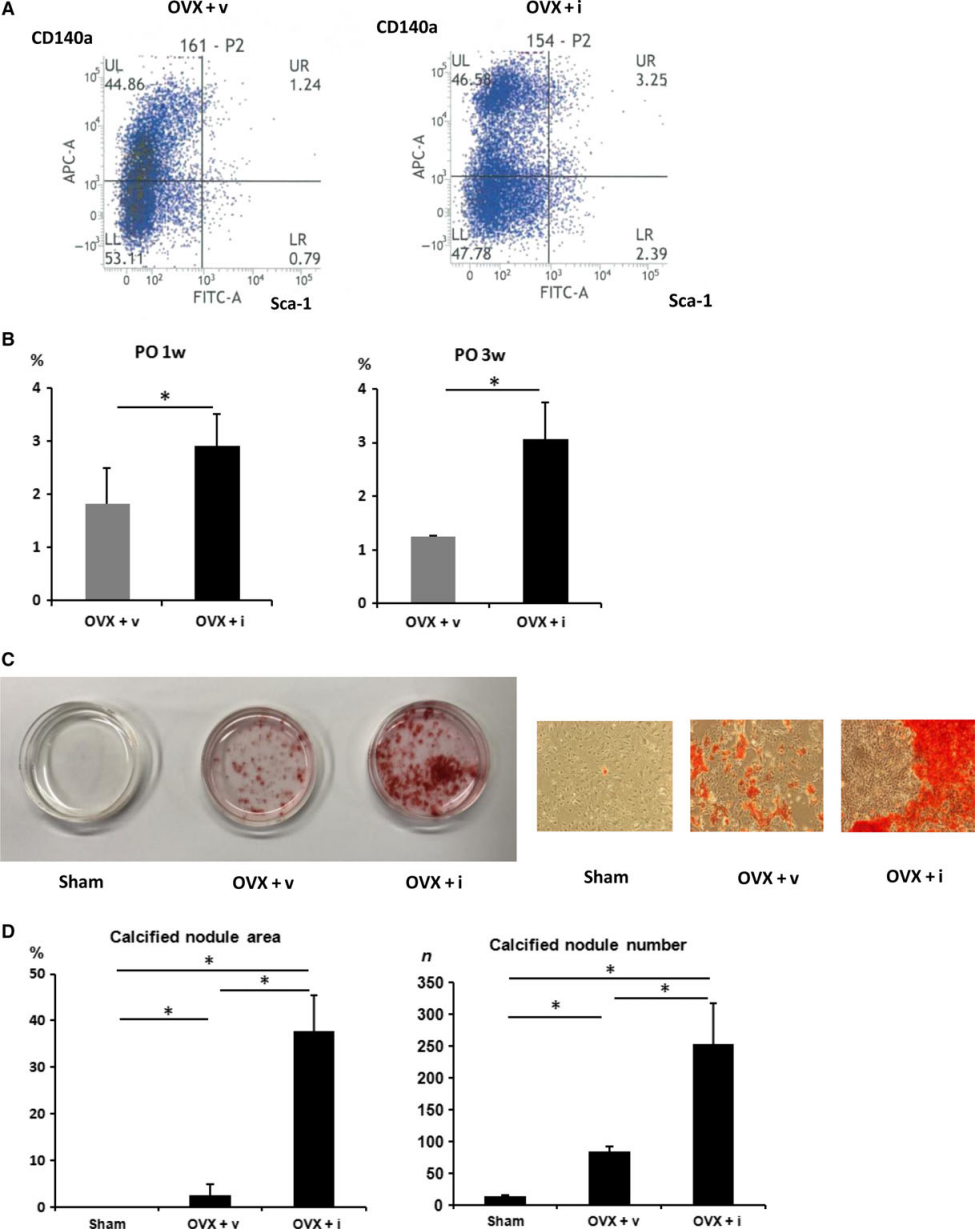
Effect of iPAI‐1 on MSC distribution and osteoblast differentiation. (A,B) iPAI‐1 administration stimulates MSC distribution in the bone marrow. (A) Bone marrow cells positive for MSC markers, such as Sca‐1 and CD140a, were significantly increased in OVX + i compared to OVX + v (B) Quantification of Sca‐1(+)CD140a(+) cells in the bone marrow cells isolated from indicated mice 1 week or 3 weeks after OVX. (C) Representative images of alizarin red staining of BMSCs isolated from indicated mice. (D) Quantification of mineralized nodule area of BMSCs isolated from indicated mice. Data represent mean ± SD for five mice/group. *P <* *0.05. OVX + v: OVX + vehicle; OVX + i: OVX + iPAI‐1.

Bone marrow stromal cells (BMSCs) differentiate into osteoblasts 16. To evaluate PAI‐1 inhibition on BMSC maintenance, bone marrow cells from OVX + v or OVX + i were examined via flow cytometry analysis. There were significantly more bone marrow cells positive for BMSC markers, such as Sca‐1 and CD140a 17, in the OVX + i group than in the OVX + v group (Fig. 4). These results indicate iPAI‐1 administration increases bone marrow MSC distribution.

To further examine the effects of iPAI‐1 on bone marrow cells, we performed a bone marrow CFU‐ob assay using cells isolated from the Sham, OVX + v, and OVX + i groups. The cells were cultured in osteoblast differentiation medium for 4 weeks. Alizarin red S staining indicated that CFU‐obs were significantly higher in the OVX + i group than those in the Sham and OVX‐v groups (Fig. 4C,D). These results demonstrate that the population of osteoblast precursor cells increased in iPAI‐1‐treated mice.

## Discussion

Here, we clearly demonstrate that iPAI‐1 prevents OVX‐induced osteoporosis. PAI‐1 is expressed by osteoblasts and osteoclasts, and its expression is stimulated by several cytokines 18, 19. Polymorphisms in *PAI‐1* are associated with the incidence of osteoporotic vertebral compression fractures 20, although serum PAI‐1 levels and functional studies for *PAI‐1* single nucleotide polymorphisms (SNPs) were not examined in this study. Several studies using PAI‐1‐deficient mice have indicated involvement of the plasminogen activator/PAI‐1 system in bone metabolism. Among them, a negative role for PAI‐1 in osteoblast differentiation has been reported 21. In our paper, iPAI‐1 treatment increased BV by stimulating osteoblasts, which is consistent with previous reports. Osteoblast differentiation was suppressed via active PAI‐1 treatment in primary osteoblasts obtained from female mice 21. Administration of iPAI‐1 stimulates expansion of osteoblast precursor cells, as demonstrated with a CFU‐ob assay. These data indicate that iPAI‐1 stimulates the commitment of BMSCs to an osteoblast lineage.

Osteoblasts are derived from BMSCs, and we show that iPAI‐1 administration increases the number of BMSCs. PAI‐1 deficiency enhances the therapeutic potential of bone marrow mononucleated cells (BMMNCs) in irradiated and nonirradiated animals via matrix metalloproteinase‐9 (MMP9) activation 22. Stem cell numbers, stem cell self‐renewal, and potentially asymmetrical stem cell division are regulated via the stem cell niche. Gelatinase B/MMP9 has been implicated in the regulation of stem cell niche behavior within the bone marrow and in the degradation of extracellular matrices within the stem cell niche, resulting in the activation and mobilization of hematopoietic stem cells. iPAI‐1 accelerate expansion of donor hematopoietic stem cells during the early stage of regeneration 23. Thus, iPAI‐1 may promote bone remodeling, probably via MMP activation‐mediated BMSC expansion, although molecular mechanisms involved in BMSC maintenance by PAI‐1 have not yet been elucidated.

Unexpectedly, iPAI‐1 partially prevented osteoclastic bone resorption in OVX mice, as shown via urinary CTX‐1 analysis and bone histomorphometry. This is the first paper demonstrating a positive role of PAI‐1 in bone resorption *in vivo*. Although osteoclasts have tPA, urokinase‐type plasminogen activator (uPA), protease nexin‐1 (PN‐1), PAI‐1, PAI‐2, and uPA receptors (uPARs) 24, several papers indicate that PAI‐1‐deficient mice do not show abnormal osteoclastic bone resorption. Further, urinary excretion of pyridinoline cross‐links, a bone resorption marker, is not affected by PAI‐1 deficiency, as shown in PAI‐1‐deficient and WT mice that underwent OVX 9. In a glucocorticoid or streptozocin‐induced osteopenia model, no effects on osteoclastogenesis were observed in PAI‐1‐deficient mice 25, 26. In addition, PAI‐1‐deficient mice show normal bone resorption in a 1,25(OH)(2)D(3)‐induced bone resorption model *in vivo* 27. The distinct differences between the results reported in the above referenced papers and our previous report are likely attributable to the method of PAI‐1 inhibition, such as gene targeting in PAI‐1‐deficient mice or pharmacological inhibition by iPAI‐1. Acute PAI‐1 inhibition by iPAI‐1 in adult mice may avoid from gene compensation, which may take place in PAI‐1 null mice. It is unclear whether the reduction in osteoclast surface in iPAI‐1‐treated OVX mice is attributable to a direct effect on the osteoclasts or a secondary genetic effect. Plasmin inhibitors such as α2‐antiplasmin are involved in OVX‐induced bone loss in mice 28. Further, α2‐antiplasmin deficiency, along with interleukin (IL)‐1β, significantly prevents elevated bone resorption following OVX 28, and α2‐antiplasmin plays a role in OVX‐induced bone loss via a mechanism that depends in part on the production of IL‐1β 28. As iPAI‐1 promotes plasmin activation like α2‐antiplasmin, some pro‐osteoclastogenesis factors might be downregulated by iPAI‐1 administration. However, the underlying molecular mechanisms still need to be elucidated.

There are several papers suggesting that PAI‐1 has a potent role in the bone metabolism of female animals 21, 26, 29. In contrast, enhanced bone callus formation is observed in a femoral bone fracture model in both male and female PAI‐1‐deficient mice 8. Here, we used a bone marrow ablation method to evaluate the function of iPAI‐1 during the rapid bone healing phase in female mice. This method has multiple advantages for evaluating rapid bone remodeling, including reproducibility and ease of quantification. Using this method, we discovered that iPAI‐1 enhances trabecular bone formation in female mice. To determine whether the gender effect of iPAI‐1 takes place in bone metabolism, we are currently planning to administer iPAI‐1 to male mice undergoing bone marrow ablation surgery. Furthermore, the effects of iPAI‐1 on streptozocin‐induced osteopenia should be elucidated.

There are several limitations to our study. First, iPAI‐1 was administered to young mice immediately after OVX. Antiresorptive agents are most effective for preserving bone mass in OVX animals when treatment is started immediately after OVX, as was done in our experiments. However, administration of parathyroid hormone, an anabolic agent for bone metabolism, to aged OVX animals leads to increased bone formation, bone mass, and bone strength 30.

As postmenopausal osteoporosis patients are elderly, the anabolic effects of iPAI‐1 on aged animal after OVX should be studied. Second, we did not evaluate bone strength, although the anabolic effects of iPAI‐1 were evident. As the goal of anti‐osteoporosis agents is to reduce the incidence of fractures, this point needs to be addressed.

In conclusion, our results suggest that PAI‐1 blockade via a small‐molecule inhibitor is a new therapeutic approach for the anabolic treatment of postmenopausal osteoporosis. The small‐molecule iPAI‐1 is a novel oral anabolic agent that may improve adherence to anti‐osteoporosis regimens.

## Author contributions

YA conceived and designed the experiments. GJ, JP, ZA, AA, KT, and YA performed the experiments. GJ, AA, YA, JP, ZA, and HO analyzed the data. DK, SS, TN, TM, and AO contributed reagents/materials/analysis tools. GJ, AA, and YA wrote the manuscript.
